# Evaluating a Digital Chronic Condition Prevention Intervention (THRIVE) in Australian General Practice: Protocol for a Mixed Methods Feasibility Study (ePREVENT-360)

**DOI:** 10.2196/83105

**Published:** 2026-05-06

**Authors:** Gillian Singleton, Elizabeth Halcomb, Andrew Bonney, Hassan Hosseinzadeh

**Affiliations:** 1 School of Medical, Indigenous and Health Sciences, Faculty of Science, Medicine and Health, University of Wollongong, Northfields Ave, University of Wollongong, Wollongong, 2522, Australia, 61 2 4221 5351; 2 School of Nursing, Faculty of Science, Medicine and Health, University of Wollongong, Wollongong, Australia; 3 School of Medicine, Faculty of Science, Medicine and Health, University of Wollongong, Wollongong, Australia

**Keywords:** digital health, chronic condition, prevention, eHealth, primary care, general practice, feasibility, patient activation

## Abstract

**Background:**

Chronic conditions are responsible for a growing burden of morbidity, mortality, and cost globally. Despite widespread recognition of the need for preventive care, general practice remains underresourced and primarily focused on treatment. Digital health interventions (DHIs) present a scalable solution to support person-centered preventive care, but evidence regarding the feasibility and acceptability of multirisk consumer-facing interventions in general practice remains limited.

**Objective:**

This study (ePREVENT-360) aims to evaluate the feasibility, acceptability, sustainability, and preliminary impact on health activation of a consumer-facing DHI, THRIVE (Tailored Health Risk Insights for Vital Empowerment) in Australian general practices.

**Methods:**

A mixed methods, pre-post feasibility study will be conducted in 5 general practices across New South Wales, Queensland, and Victoria. Adult consumers aged 30 to 65 years will use the THRIVE digital platform to receive chronic condition risk assessments, health scores, and action plans. Quantitative data will include engagement metrics, surveys, and chronic condition risk scores. Qualitative semistructured interviews with consumers and clinicians will provide data about acceptability, engagement, and sustainability. Quantitative data will be analyzed using descriptive and multilevel regression methods, while qualitative data will be analyzed thematically.

**Results:**

The study has secured funding in 2024 through an Australian General Practice Research Foundation and Hospital Contribution Fund of Australia Research Foundation Health Services Research Grant. Consumer recruitment commenced in December 2025. Recruitment of the 5 participating general practices was completed in March 2026. As of April 2026, all clinician preintervention interviews have been completed, and consumer recruitment has commenced, with 25 consents obtained. Data collection is ongoing, with follow-up expected to be completed by December 2026. Outcomes will inform the iterative refinement of interventions and future trial designs to assess effectiveness.

**Conclusions:**

This study will address a key evidence gap in the digital prevention space by evaluating the feasibility, acceptability, and sustainability of a multicondition DHI embedded in general practices. The findings will support the development of a larger adaptive controlled trial and inform future implementation.

## Introduction

### Background and Rationale

Chronic conditions, including cardiovascular disease, type 2 diabetes, and common cancers, contribute to a substantial and growing global health and economic burden [1-3]. These conditions arise from complex interactions between modifiable (eg, smoking, inactivity, and poor diet) and nonmodifiable (eg, age, ethnicity, and family history) risk factors [4]. Importantly, over 17 million premature deaths each year are attributable to preventable causes [1,2], highlighting the critical role of primary and secondary prevention in reducing disease burden and improving population health outcomes.

While the cost-effectiveness and long-term benefits of prevention are well established, many health systems continue to struggle to deliver equitable, timely, and comprehensive preventive care—particularly within general practice [5-7]. General practice serves as the frontline of health care, encompassing health promotion, early detection and intervention, and the management of acute and chronic conditions [8]. In Australia, despite national policy commitments to preventive health [9]; systemic barriers, such as fragmented services and inadequate financial incentives for preventive activities; and chronic underinvestment in general practice continue to impede the implementation of effective prevention models [10-13].

Participation in preventive services remains suboptimal in Australia, with only 52% of eligible women participating in breast cancer screening [14] and only 42% of eligible adults undertaking bowel cancer screening [15]. Routine recording of risk factor data for common chronic conditions, such as cardiovascular disease and type 2 diabetes, is also inconsistent. This limits the feasibility of proactive risk identification, with data recorded for only 49% and 24% of the population, respectively [16,17]. These gaps underscore the need for scalable, system-wide strategies to increase access to and engagement with preventive care. Digital health interventions (DHIs) are a promising avenue to strengthen preventive care delivery, yet their acceptability, engagement, and feasibility in real-world general practice settings remain underexplored.

### Challenges to Effective Prevention at the Individual Level

Supporting sustained improvements in lifestyle risk factors is a significant challenge, with effective and scalable solutions yet to be fully established. High health literacy [18-20] and patient activation [21-29] have been strongly associated with improved lifestyle risk profiles. Patient activation is an individual’s knowledge, skills, and confidence in managing their health [30,31]. Emerging evidence suggests that patient activation is most strongly associated with improved health behaviors, increased participation in screening and preventive care, and better overall health outcomes than health literacy alone [21,22,24]. Despite its potential, the role of activation in the primary prevention of chronic conditions remains underexplored [21,22,25,29]. Thus, identifying and evaluating effective strategies to enhance and sustain patient activation in general practice is a pressing area for future research.

### Digital Health and Emerging Opportunities

The COVID-19 pandemic catalyzed the rapid adoption of DHIs [32]. These interventions are diverse, ranging from automated SMS reminders to complex interventions integrated with electronic health systems that use natural language processing, machine learning, and artificial intelligence [33,34]. DHIs offer considerable potential to collect and track real-time individual health data and to personalize health promotion, health education, and behavior change content to individual needs and risk profiles [35]. Sustained behavior change addressing lifestyle risk factors is an essential aspect of effective prevention, and DHIs are recognized to be an effective vehicle for persuasion compared to human-delivered interventions [36]. Despite this promise, most existing digital health research has centered on narrow, condition- or risk factor–specific applications—such as behavior change interventions (eg, weight loss [37], smoking cessation [38]), condition management (eg, diabetes self-monitoring [39-41]), or clinician-facing tools (eg, decision support or telehealth [42,43]). In contrast, there is comparatively little evidence examining the role of consumer-facing DHIs designed to deliver comprehensive primary and secondary prevention [39,40,42], meaning the potential of these technologies to enable real-time monitoring of population-level health indicators remains unrealized [39,42,44].

Despite the substantial contribution of social, environmental, and behavioral factors to the risk of developing common conditions, these indicators are not routinely captured or used meaningfully to enable precision prevention and monitoring [44]. Persistent barriers to digital health adoption, including a lack of trust, particularly among groups with low digital literacy and overlapping social determinant risk factors, remain to be addressed [33,45]. There are critical gaps in the current landscape, including the need for rigorously testing digital interventions to empower consumers to understand and act on their risk in general practice. Furthermore, understanding whether low health activation functions as an obstacle to engagement with digital interventions is an important research priority. Addressing these gaps is essential to developing scalable, sustainable models of preventive care [46].

### Objectives

The primary objectives of ePREVENT-360 are to assess the feasibility of the THRIVE (Tailored Health Risk Insights for Vital Empowerment) digital intervention for adults in Australian general practices by (1) examining factors that influence clinician and consumer engagement, (2) evaluating the intervention’s acceptability, and (3) exploring perceptions of the intervention’s sustainability. The secondary objective is to assess the preliminary effectiveness of THRIVE on consumer activation, self-rated health, and chronic disease risk scores.

## Methods

### Study Design

This protocol is reported according to the SPIRIT (Standard Protocol Items: Recommendations for Interventional Trials) 2025 guidelines (Checklist 1) [47]. The ePREVENT-360 study is a mixed methods, pre-post feasibility trial designed in accordance with the UK Medical Research Council (MRC) framework [48] for evaluating complex interventions. The study will be guided by 2 complementary theoretical models to inform the assessment of user acceptability: the extended unified theory of acceptance and use of technology 2 (UTAUT2) [49,50] and the theoretical framework of acceptability [51].

### Settings

The study will be conducted in 5 general practices in New South Wales, Queensland, and Victoria, Australia. Practices will be recruited through local primary health care organizations (primary health networks), practice-based research networks, relevant professional organizations, and snowballing. Practices with a minimum of 5 general practitioners will be prioritized to ensure sufficient organizational capacity to support study onboarding, patient recruitment, and study processes. Larger practices typically have more stable administrative support, larger patient volumes, and established prevention and management workflows, making them more suitable for inclusion.

Practices will be purposively selected to ensure geographic diversity across metropolitan, regional, and rural locations. Each participating practice will be asked to nominate 1 general practitioner or general practice nurse to act as the study champion. This champion will receive comprehensive study information and lead the practice’s involvement. General practices will provide informed consent, including an agreement to promote study participation to eligible patients and clinicians.

### Participants

#### Consumer Sampling and Recruitment

A convenience sample of consumers will be recruited from participating practices. Eligible consumers will be adults aged 30 to 65 years, who can speak English and have regular access to an internet-enabled digital device, such as a smartphone, tablet, or computer. This age range was selected to maximize preventive potential, targeting individuals most likely to benefit from lifestyle changes and screening engagement before the onset of chronic conditions.

Each practice will recruit consumer participants over a 3-month recruitment period. The recruitment process will be multifaceted. Primarily, brochures or posters with QR codes will be displayed in waiting and consulting rooms, allowing individuals to access information via their mobile phones while they are waiting. This QR code links to a webpage where people can register their interest, receive study information, and complete the consent form. Additionally, study information will be disseminated via practice newsletters and/or emails if available. To minimize burden on the participating practices, clinicians are not expected to recruit consumers; however, individual clinicians can opportunistically encourage consumers to consider participation.

#### Clinician Sampling and Recruitment

All general practitioners and general practice nurses from participating practices will be invited to take part in the preintervention and postintervention interviews and the postintervention survey. Clinician participation will be entirely voluntary. Study information, including a QR code link to the survey or consent form, will be emailed to potential participants by the practice. Additionally, a QR code linking to the survey or consent form will be displayed in the practice staff areas.

### Sample Size

#### Consumer Participants

As a feasibility study, this trial is not powered to detect statistically significant differences in outcomes. Instead, the sample size was determined to allow for the estimation of key feasibility indicators with acceptable precision [52]. If approximately 50% of participants who consent complete the THRIVE health assessment, with a 10% margin of error at a 95% CI, a minimum sample size of 187 consumers will be required.

In line with previous general practice research [53-57], a cluster adjustment was applied to the sample calculation using an intracluster correlation coefficient of 0.05 [53-57]. With an anticipated 5 participating practices and a design effect of 1.95, allowing for an estimated 15% dropout rate, the adjusted sample size requirement will be 215 consumers. Therefore, each practice is expected to recruit at least 43 participants.

#### Clinician Participants

All clinicians from all participating practices will be invited to participate. The clinician sample size will be pragmatic and guided by the study objectives, given the outcomes being measured.

### Intervention

#### Intervention Development

THRIVE was developed through an iterative design process, drawing on principles of co-design [58,59], contemporary behavior change theory, preventive care guidelines, persuasive system design, and principles from nudge theory [60]. In alignment with evidence highlighting the value of motivational communication and self-monitoring [42,43,61,62], nudge design-based principles, such as timely prompts, simplified decision pathways, concise feedback, and guideline-aligned options, are embedded.

Meaningful engagement with community members occurred throughout development. This iterative consultation shaped the platform’s functionality, content clarity, user pathways, notification timing, and overall usability. These refinements aimed to enhance engagement, reduce respondent burden, and ensure that behavioral prompts and decision pathways were intuitive and fit for users’ real-world needs. All consumer-facing content is intentionally designed to be practical and easy to follow, written at a year 7 reading level to maximize comprehension and accessibility [63]. THRIVE is currently only available in the English language. Establishing technical feasibility, core functionality, acceptability, and engagement is important before multilingual versions are developed.

The intervention’s theoretical foundation incorporates the transtheoretical model [64] and the health action process approach [65] to support goal-setting, self-efficacy, and sustained behavior change across different stages of readiness. Preventive action plans and educational content are aligned with contemporary Australian clinical guidelines, including the Royal Australian College of General Practitioners Red Book [66], Cancer Council [67], National Heart Foundation [68], and Kidney Health Australia [69] guidelines. THRIVE follows a continuous update policy, in which content is revised promptly in response to updates to national guidelines or new evidence. All changes are documented using structured version control procedures (Multimedia Appendix 1 [66,68-91]).

THRIVE generates personalized lifetime chronic condition risk estimates using a composite lifetime modeling framework. This model synthesizes demographic variables, behavioral risk factors, family history, anthropometric measures, and selected clinical data based on evidence from Australian and international epidemiological studies. Inputs and effect sizes are derived from hazard ratios, attributable risk fractions, and pooled effect estimates from meta-analyses and high-quality cohort data. Construct validation has been undertaken for risk estimates that have existing short-term (typically 5-year-risk) calculators by benchmarking THRIVE-generated risk scores against these tools, including the Australian Type 2 Diabetes Risk Assessment Tool [70], the Australian Cardiovascular Risk Score [71], and STOP-BANG for sleep apnea [92]. Validation analyses demonstrated moderate (STOP-BANG) to near-perfect (Australian Type 2 Diabetes Risk Assessment Tool) construct alignment with these established tools, and all recommendations are consistent with Australian clinical guidelines [66-69] (Multimedia Appendix 1 [66,68-91]).

Findings from this study, including quantitative engagement metrics and qualitative acceptability data, will inform subsequent model refinement to enhance usability in practice, ensure clinical appropriateness, and integrate into clinical workflows before a future large-scale trial.

#### Intervention Content and User Experience

THRIVE guides users through lifetime chronic condition risk assessments, facilitates goal setting, and tracks and reinforces progress through behavioral “nudges.” The intervention is largely automated and can be used independently by consumers. Once consumer participants have provided consent and completed the preintervention survey, they receive a personalized email link providing access to the THRIVE platform. The online assessment guides users through a dynamic, user-friendly series of questions tailored to individual responses. Questions incorporate 130 validated risk and protective factors, and participants receive real-time feedback to maintain motivation and minimize attrition.

After completing the assessment, participants access a personalized dashboard that presents lifetime risk estimates for 23 chronic conditions, including cardiovascular disease, metabolic conditions, osteoporosis, sleep apnea, various cancers, eye and kidney disease, and an estimated “medical age.” The medical age score reflects the impact of lifestyle choices on microlife calculations (a microlife is a statistical unit representing 30 minutes of life expectancy gained or lost due to daily habits). Color-coded indicators highlight key contributors to risk. These risk estimates and the medical age-metric personalize lifetime risk estimates. Risk estimates and the “medical age” metric are not diagnostic tools and are intended solely for promoting health education and engagement. Multimedia Appendix 1 [66,68-91] provides a summary of all variables included in THRIVE’s risk-modeling framework, as well as the guideline sources informing action-plan content.

The platform translates risk estimates into actionable guidance using rule-based logic aligned with Australian clinical guidelines [66-69]. After viewing their dashboard, participants can access an automated action plan that provides tailored lifestyle and medical screening recommendations aligned with current Australian guidelines. Recommendations are presented using nudge-informed design principles that simplify decision-making while preserving user autonomy.

Consumers can also access a clinician report that consolidates risk estimates, key health indicators, and recommendations, which they can choose to share with their general practitioner or general practice nurse to facilitate preventive-care discussions. This report also identifies Medicare Benefits Schedule items that the consumer may be eligible to receive to promote access to care. However, clinicians retain full discretion regarding consumer eligibility and the appropriateness of any care activity and associated Medicare Benefits Schedule claims.

**Figure 1.**
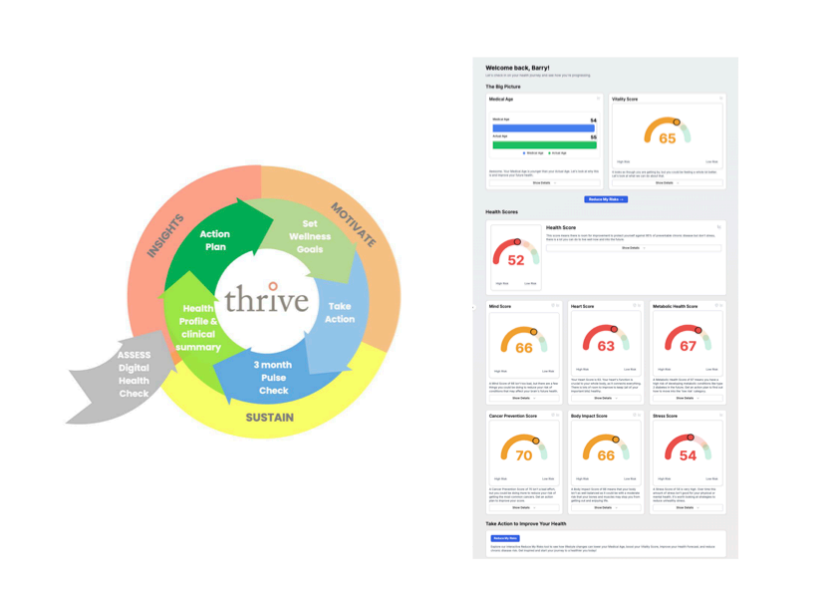
THRIVE dashboard image and platform overview. THRIVE: Tailored Health Risk Insights for Vital Empowerment.

Consumers are encouraged to set health goals and may choose to integrate data from wearable devices. They can also complete a quarterly “pulse check,” a brief reassessment that updates their health data, provides refreshed risk calculations, and tailors action plans. To support ongoing engagement, participants retain continuous access to their dashboard and can revisit it at any time to review progress or modify goals. Optional behavioral prompts and reminders are delivered to further support sustained use. Figure 1 illustrates the THRIVE dashboard and platform overview.

#### Integration With General Practice Workflow

As a prototype, THRIVE currently operates as an externally hosted digital platform that is not integrated into electronic medical record (EMR) systems. To align with clinical workflows, patients access study information through QR codes in the waiting room, enabling them to complete the risk assessment before, during, or between appointments. Clinicians will still retain the ability to refer consumers whom they feel may benefit.

As a consumer-facing intervention, THRIVE empowers consumers to share their study involvement with clinicians through the clinician report, should they wish to prompt discussion about preventive care. Behavioral nudges are provided directly to consumers by THRIVE, without the need for clinician input. This direct link to consumers seeks to support general practitioner and general practice nurse workflows by providing health education, personalized recommendations, and ongoing encouragement.

Through the provision of tailored health information and health promotion education directly to consumers, THRIVE aims to improve the efficiency of preventive care delivery, thus supporting general practitioner and general practice nurse workflows. This feasibility phase will explore how consumers and clinicians prefer THRIVE to interface with general practice, and the findings will inform future co-design and staged EMR integration.

### Data Collection

#### Overview

**Figure 2.**
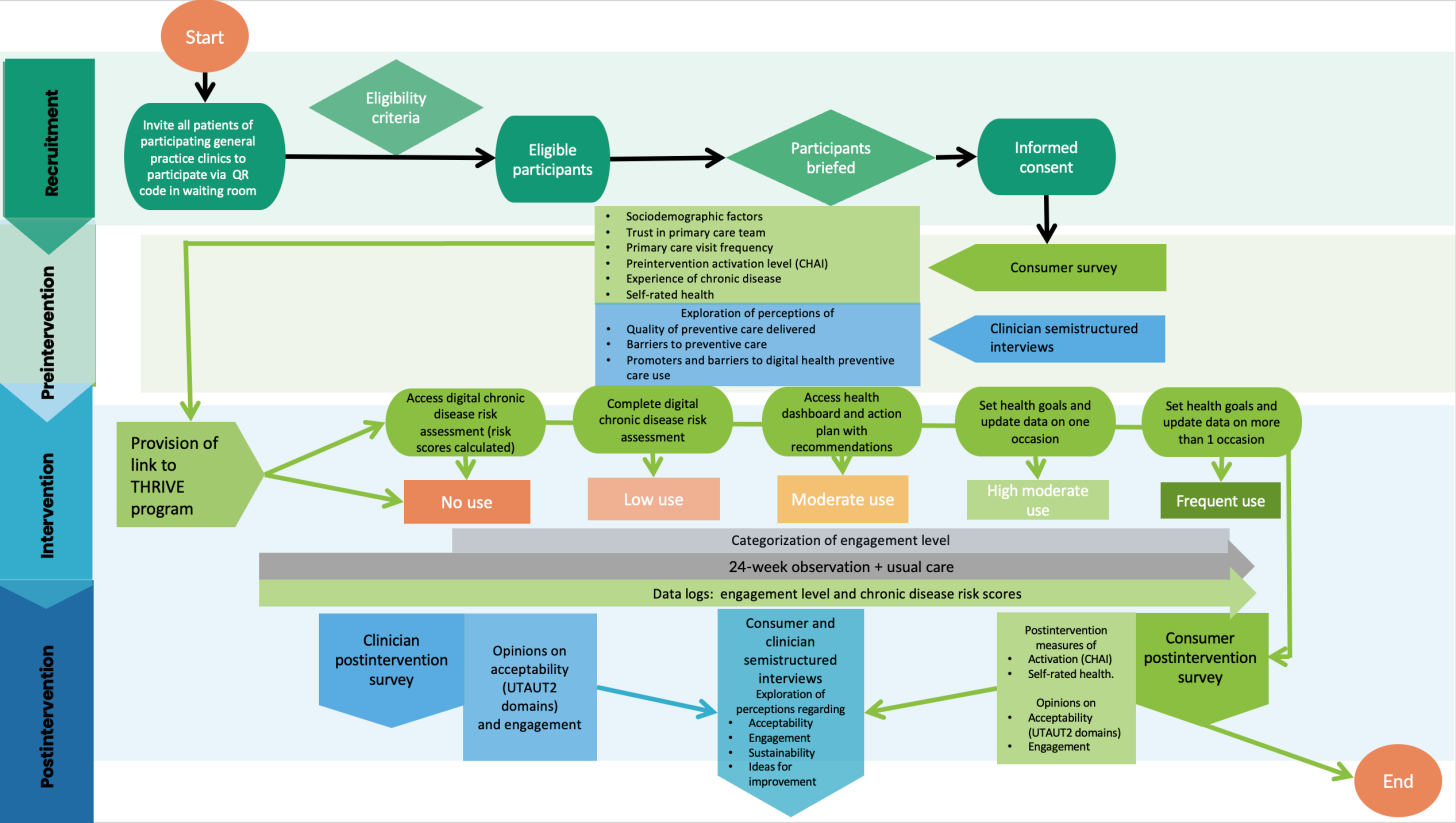
ePREVENT-360 study flowchart. CHAI: Consumer Health Activation Index; THRIVE: Tailored Health Risk Insights for Vital Empowerment; UTAUT2: extended unified theory of acceptance and use of technology 2.

The recruitment period will span approximately 3 months, with follow-up data collection and analysis concluding around 9 months from the start of recruitment. Data will be collected from consumer and clinician participants across multiple time points. A mixed methods approach will be used, with qualitative insights complementing and adding depth to quantitative data (Figure 2).

#### Consumer Data Collection

Quantitative data will be collected via self-administered online surveys at baseline and 6 months post enrollment. These surveys will assess outcomes related to acceptability, engagement, and early indicators of effectiveness, including activation and self-rated health. The estimated total time commitment for consumer participants is 75 minutes over 6 months, including participation in surveys, digital assessments, and optional interviews.

Engagement data will be passively collected through the THRIVE platform, including metrics such as login frequency, dashboard views, goal setting, action plan access, and responses to health reminder prompts. Following the postintervention survey, participants will be invited to opt-in to subsequent interviews by providing their contact details. As part of this process, participants will self-report their level of intervention engagement (eg, high, medium, low, or none), to guide sampling stratification. This process ensures that codified (controlled) and identifiable (protected) data are handled separately.

Approximately 18 to 20 consumer interviews (up to 4 per clinic) will be conducted via videoconference, audio-recorded, transcribed verbatim, and complemented by researcher field notes. A qualitative descriptive approach will be used to explore perceptions of intervention acceptability, sustainability, impact, and opportunities for improvement. Final numbers will be guided by data sufficiency. The semistructured interview guide is included in Multimedia Appendix 1 [66,68-91].

#### Clinician Data Collection

Preintervention and postintervention clinicians will be invited to participate in semistructured interviews. These interviews will explore key feasibility domains such as acceptability, sustainability, perceived impact, and optimization strategies. Approximately 8 to 10 clinicians (2 per practice) will be interviewed via videoconference, with sampling guided by saturation. Interviews will be guided by qualitative descriptive methodology. Interview guides are included in Multimedia Appendix 1 [66,68-91]. The time commitment for clinicians will range from 10 to 40 minutes, depending on their level of participation across survey and interview components.

Clinicians will also complete a postintervention survey. The survey includes 15 items rated on a 7-point Likert scale and captures data on intervention acceptability, perceived usefulness, and ease of implementation, impact on workflow, barriers, and summary of use.

### Outcomes

#### Overview of Outcome Measures

The primary focus of this study is feasibility, which will be assessed through consumer and clinician engagement, acceptability, and perceived sustainability (Table 1). A secondary outcome is preliminary effectiveness, evaluated through changes in consumer activation, self-rated health, and disease risk scores.

**Table 1.** Overview of outcome measures.

Outcome and components	Data collection tool	Participants	Collection timeframe
Primary outcome (feasibility)			
Engagement	Usage logs from THRIVE platform	Consumers	Observation period
Acceptability	UTAUT2^a^ survey questions [93-95]	Consumers and clinicians	Postintervention
Sustainability	Semistructured interviews	Consumers and clinicians	Postintervention
Secondary outcomes (effectiveness)			
Consumer activation	CHAI^b^ survey questions [96]	Consumers	Preintervention and postintervention
Self-rated health	Single-item Likert Scale survey question	Consumers	Preintervention and postintervention
Calculated disease risk scores	AUSDRISK^c^ [70] and AusCVDRisk^d^ [71] scores calculated from THRIVE data	Consumers	Observation period

a UTAUT2: extended unified theory of acceptance and use of technology 2.

b CHAI: Consumer Health Activation Index.

c AUSDRISK: The Australian Diabetes Risk Score.

d AusCVDRisk: The Australian Cardiovascular Disease Risk Score.

The consumer baseline survey will also capture factors that may influence engagement with the intervention, including sociodemographic characteristics, perceived disease threat, and trust in the therapeutic relationship. Perceived disease threat will be measured using 4 validated items from the UTAUT2 framework [93], rated on a 7-point Likert scale ranging from “strongly disagree” to “strongly agree” (⍺=0.817). Trust in the therapeutic relationship will be assessed using the Trust in Physician Short-Form Scale [97]. These baseline variables will form the a priori covariate set for exploratory quantitative analyses examining predictors of engagement with the THRIVE intervention and potential signals of change in secondary outcomes. They will also be used to inform question refinement for the postintervention interviews, ensuring representation of diverse experiences and perspectives across engagement levels.

#### Engagement

Engagement will be measured using THRIVE usage logs, which automatically record participants’ interactions with the platform. Based on these behaviors over the 6-month study period, participants will be classified into 1 of 5 engagement levels (Figure 2). Individuals who consent but do not initiate or complete the risk assessment will be classified within the “no use” engagement category. This group will be included in follow-up surveys and purposive qualitative sampling to capture barriers to engagement.

#### Acceptability

Consumer acceptability will be assessed in the postintervention survey using items from 8 of the UTAUT2 domains: performance expectancy, effort expectancy, social influence, facilitating conditions, hedonic motivation, trust, habit, and behavioral intention Multimedia Appendix 1 [66,68-91] [49,50,93,98]. The UTAUT2 has been widely applied to evaluate the adoption of consumer-facing technologies [50,93,99,100]. It has demonstrated strong internal consistency (Cronbach α range=0.637‐0.902 across domains) [93] and high reliability to evaluate acceptability (range ⍺=0.837‐0.899) [95]

Items are rated on a 7-point Likert scale, from 1 (“strongly disagree”) to 7 (“strongly agree”), with higher scores indicating greater acceptability. Six questions across 5 domains are shared between the 2 groups, with additional questions tailored to context-specific relevance depending on engagement levels. For example, in the performance expectancy domain, users respond to “I found the THRIVE program to be useful,” while nonusers respond to “I know what to do to stay healthy so I didn’t need this program.”

User participants will complete 18 items across the 8 UTAUT2 domains. Acceptability outcomes will be reported using the following 2 complementary metrics [51]: (1) the mean acceptability score across domains with scores of more than 4 considered indicative of acceptability (ie, responses in the “somewhat agree” to “strongly agree” range) and (2) the proportion of participants whose individual mean scores meet or exceed the threshold, with 70% or more representing a benchmark for acceptable uptake. Together, these metrics provide insights into both the overall perceived acceptability and the consistency of this perception across participants.

Nonuser participants will complete 12 items across 7 relevant domains (excluding “habit,” which is not applicable to those who did not engage with the intervention). The primary purpose of this analysis is to explore the domain-specific barriers to acceptability. First, item-level responses of more than 4 will be interpreted as indicating a potential concern within a specific domain. Second, we will report the proportion of nonusers whose mean domain scores are more than 4 to reflect broader patterns of perceived difficulty or lack of relevance.

These acceptability thresholds align with typical group-level acceptability criteria in other digital health evaluations [101,102] and were established a priori (Table 2).

**Table 2.** Consumer extended unified theory of acceptance and use of technology 2 (UTAUT2) acceptability domains and threshold values.

Consumer participant acceptability measures		Users (number of items)^a^	Nonusers (number of items)^b^
UTAUT2 domains	Cronbach α		
Performance expectancy	0.833	3	1
Effort expectancy	0.902	2	4
Social influence	0.850	2	2
Facilitating conditions	0.773	3	1
Hedonic motivation	0.807	2	1
Habit	0.637	1	—^c^
Trust	0.830	4	2
Behavioral intention	0.901	1	1

a Acceptability threshold: mean score >4 and ≥70% participants meeting this threshold indicates acceptability.

b Barrier indicator: mean score >4 and ≥70% of participants meeting this threshold indicates a barrier to use.

c Not applicable.

Clinician acceptability will be assessed in the postintervention survey using 15 items drawn from 6 relevant UTAUT2 domains: performance and effort expectancy, social influence, facilitating conditions, trust, and behavioral intent Multimedia Appendix 1 [66,68-91]. These items reflect perceptions of program usefulness, its impact on care efficiency and/or remuneration, perceived barriers to consumer use, and the use of the clinician summary reports. Four survey items are negatively worded and will be reverse scored before analysis.

Acceptability outcomes will be reported using 2 complementary metrics, consistent with the approach for consumer participants. First, an overall mean acceptability score will be calculated across the 15 items. A mean score of more than 4 will be interpreted as indicating acceptability, representing a tendency toward “somewhat agree” or higher (Table 3). Second, we will report the proportion of clinicians whose individual mean score is more than 4, with 70% or more of participants meeting this threshold considered evidence of general acceptability [51]. These dual metrics allow both group-level and individual-level interpretation of findings. Additionally, domain-specific mean scores and proportions will also be calculated to explore patterns across the 6 UTAUT2 domains.

**Table 3.** Clinician acceptability domains and cutoff scores.

UTAUT2^a^ domains	Number of items^b^
Performance expectancy	7
Effort expectancy	3
Social influence	1
Facilitating conditions	2
Trust	1
Behavioral intention	1

a UTAUT2: extended unified theory of acceptance and use of technology 2.

b Acceptability threshold: mean score>4 and ≥70% participants meeting this threshold indicates acceptability.

#### Sustainability

Perceptions of sustainability will be evaluated through semistructured individual interviews with consumers and clinicians. These qualitative descriptive interviews will explore user experiences, perceived barriers and enablers to continued use, and suggestions for long-term implementation and integration into clinical workflows.

#### Preliminary Effectiveness

The secondary outcomes are included to explore early signals of the intervention’s potential effectiveness and to inform future trial design. These outcomes include changes in consumer activation, self-rated health, and calculated disease risk scores. Consumer activation will be assessed using the 10-item Consumer Health Activation Index (CHAI), a validated measure of an individual’s knowledge, skills, and confidence in managing their health [96]. CHAI has demonstrated high internal reliability and predictive validity for health behavior change [27,96,103]. Self-rated health will be measured via a single-item rated on a 5-point Likert scale, which is widely used as a proxy for general health status [104]. Chronic condition risk scores will be calculated from data within the THRIVE platform at baseline and at 6 months postenrollment.

#### Progression Criteria

Consistent with contemporary guidance for feasibility studies of complex and digital health interventions, such as the UK MRC framework [48], a flexible progression framework will be used rather than a binary go or no-go decision. Feasibility outcomes (engagement and acceptability) will be interpreted alongside qualitative findings to guide iterative refinement of the intervention prior to a future effectiveness trial.

A 3-level progression approach will be applied as follows:

- Proceed: primary feasibility indicators meet predefined thresholds, and qualitative data suggest overall acceptability and workflow fit.
- Proceed with modifications (expected primary pathway): one or more indicators fall below the threshold, or qualitative findings identify modifiable barriers. Findings will inform targeted refinements to the intervention, implementation strategies, or study procedures before advancing to a powered trial.
- Do not proceed without redesign: multiple feasibility indicators fall below the threshold, and qualitative data indicate fundamental concerns that cannot be addressed through minor modifications.

This approach is consistent with the study’s purpose—to inform iterative refinement and future trial design rather than to provide definitive go or no-go criteria.

### Data Management

The THRIVE dashboard and clinician summary report are visible only to the consumer participant through their secure, personalized link. No identifiable or dashboard data are shared with clinicians or transferred to general practice systems. Only the consumer participant can choose to share their outputs or the clinician report.

Identifiable consumer participant information (first name and email address) will be collected solely to link preintervention and postintervention data. These identifiers will be collected within the secure University of Wollongong Qualtrics survey platform and stored separately from other study data. An independent research officer will assign each participant a unique study code to enable matching of deidentified data across time points. The linkage file containing names, emails, and study codes will be stored separately from all study datasets and will be accessible only to the independent research officer. Once the linkage process is complete, only deidentified datasets will be stored in the University of Wollongong Microsoft Teams environment, consistent with institutional data governance policies. For the interviews, the interviewer will have access to participants’ names and email addresses for scheduling and conducting interviews. Audio recordings and transcripts will be stored securely in the Microsoft Teams environment, consistent with institutional data governance policies. The research officer will deidentify transcripts and assign interview codes. These codes will be independent of those used for the quantitative data. Only deidentified data will be used for analysis.

### Data Analysis

#### Quantitative Data

To ensure analytical independence, all quantitative data extraction and cleaning will be performed exclusively by an independent research officer, who has no affiliation with In2Health Solutions. The PhD candidate will not access any study dataset until the statistical analysis plan (SAP) has been finalized, independently reviewed, and approved by the supervisors (HH, EH, and AB). The SAP will be preregistered and archived on the Open Science Framework prior to data extraction. Analyses will be conducted by the PhD candidate and independently verified by the research officer, with academic oversight provided by the supervision team. All analytical code will be version-controlled, and the SAP will contain the IBM SPSS (Statistical Package for Social Sciences) syntax for multilevel regression models, missing data procedures, and sensitivity analyses.

#### Primary Outcome Analyses (Feasibility End Points)

Descriptive statistics will be calculated for all variables, including frequencies for categorical data and means or medians for continuous variables. Engagement levels will be examined in relation to participant characteristics using nonparametric methods, including the Kruskal-Wallis *H* test and the Spearman rank correlation. Acceptability scores will be summarized for each domain of the UTAUT2 [49,98] and compared between user subgroups.

The proportion and patterns of missing baseline and follow-up data will be documented as part of the feasibility assessment. Given the descriptive nature of feasibility end points, no imputation will be undertaken. For baseline acceptability data, incomplete responses will be excluded from domain-level mean score calculations using listwise deletion [105]. Missing usage log data will be interpreted as nonengagement rather than missingness.

As a secondary exploratory analysis, multilevel logistic regression models will be used to examine predictors of engagement, with practice included as a random intercept to account for clustering.

#### Secondary Outcome Analyses

Analyses of consumer activation, self-rated health, and chronic disease risk scores will be exploratory and are not intended to test hypotheses or infer causal effects. In the absence of a comparator group, these models will be used solely to identify potential signals of change and to inform outcome selection and sample size calculations for a future effectiveness trial.

To minimize analytic flexibility, covariates have been prespecified a priori and include age, gender, education, employment status, postcode (as a proxy socioeconomic indicator), baseline activation (CHAI), perceived disease threat, and trust in the therapeutic relationship. The practice site will be modeled as a random intercept to account for clustering at the practice level.

The proportion and patterns of missing data will be documented. If missingness exceeds 5%, multiple imputation using predictive mean matching will be applied to secondary outcome models [106]. Listwise deletion will be used for missing baseline covariates where required.

Consistent with feasibility study guidance, no formal adjustment for multiplicity will be undertaken. Effect estimates and 95% CIs will be reported descriptively to explore the direction and magnitude of potential change, without inferential interpretation of statistical significance. Sensitivity analyses for the exploratory multilevel models will include re-estimation under alternative intracluster correlation coefficient assumptions to assess the robustness of clustered models.

### Qualitative Data

Qualitative interview transcripts will be uploaded to NVivo (Lumivero) and analyzed using the Braun and Clarke 6-phase approach [107]. After immersing themselves in the data, the PhD candidate and research officer will code a sample of transcripts independently. Coding discrepancies will be resolved through discussion or the involvement of a supervisor (EH). Reporting will follow the COREQ (Consolidated Criteria for Reporting Qualitative Research) guidelines (Checklist 2) to ensure transparency [108].

### Ethical Considerations

The ePREVENT-360 study has received approval from the University of Wollongong Human Research Ethics Committee (2024‐274). Participation is entirely voluntary, and choosing not to participate will not affect consumer access to care or clinicians’ relationships with the University of Wollongong or their practice. Informed consent will be obtained via an online form that clearly outlines the study’s purpose, the voluntary nature of participation, and assurances regarding privacy and confidentiality.

The intervention is low-risk and designed to benefit participants by providing personalized health insights and support for preventive health behaviors. The intervention content has been carefully worded to minimize the risk of distress, particularly when communicating chronic condition risk. Participants are encouraged to consult their general practitioner or general practice nurse regarding any concerns, ensuring continuity of care. Support resources will be provided, especially regarding questions about family history of serious illness. The research team will monitor for unintended effects, and any issues raised by participants will be documented and reviewed. Participant compensation is aligned with the National Health and Medical Research Council’s [109] guidance on participant payments; gift cards will be provided to acknowledge participants’ contributions without inducing participation. Consumer participants will receive an Aus $50 (US $39) gift card upon completion of all study measures and Aus $30 (US $21) for partial completion. Clinician and consumer interview participants will receive an Aus $80 (US $57) gift card.

Access to all study data will be restricted to authorized researchers through password-protected, role-based permissions. Analysis scripts and deidentified datasets will be version-controlled, with a full audit trail maintained to ensure reproducibility. All data will be retained in the Microsoft Teams environment for 5 years before being destroyed in accordance with university policy.

Project governance is provided by a supervisory committee of 3 experienced and independent academic supervisors (HH, EH, and AB), who will oversee all elements of the study design, execution, analysis, and reporting. To ensure rigor and minimize bias, data integrity will be assessed through access records, date stamps, and reproducible analytic processes. The supervision team (HH, EH, and AB) will critically review all analyses. Given the feasibility nature of this study and the absence of high-risk intervention or large-scale recruitment, a formal data monitoring committee will not be convened.

### Protocol Modification Plan

Any modifications to the study protocol that may impact the conduct of the study or its scientific validity (eg, changes to eligibility criteria, outcomes, analyses, or procedures) will be submitted for review and approval by the Human Research Ethics Committee prior to implementation. All amendments will also be communicated to the funding bodies. A summary of any protocol changes will be documented and reported in any resulting publications and the trial registration.

## Results

This study is registered with the Australian New Zealand Clinical Trials Registry (12624001174572). The study was funded in 2024 through the Australian General Practice Research Foundation and Hospital Contribution Fund of Australia Research Foundation Health Services Research Grant. Recruitment of the 5 participating general practices was completed in March 2026. As of 17 April, 2026, all clinician preintervention interviews have been completed, and consumer recruitment has commenced, with 25 consents obtained. Data collection is ongoing, with follow-up expected to be completed by December 2026. Outcomes from this study will inform the iterative refinement of the THRIVE intervention and the design of a future effectiveness trial.

## Discussion

This protocol outlines the methodology and methods of the ePREVENT-360 study, which aims to evaluate a multiconditional, consumer-facing DHI—THRIVE—designed to support chronic condition prevention within Australian general practice. By delivering personalized risk assessments and enabling goal setting and action planning, THRIVE offers a novel, scalable strategy to help close persistent gaps in preventive health engagement and delivery [9,14-17]. This study prioritizes key feasibility measures, including consumer and clinician acceptability, engagement, and potential for integration into routine general practice [33,42,48,51,61].

There is growing recognition that scalable, person-centered DHIs have the potential to enhance consumer activation and promote earlier identification and management of chronic condition risk [35,36,39-41,43]. While such tools have shown promise, particularly in promoting individual behavior change [37,38], relatively few have been developed specifically for comprehensive primary prevention or designed for integration within general practice workflows [39,40,42]. Critical gaps remain regarding feasibility evaluation, particularly around consumer and clinician engagement, acceptability, and the long-term sustainability of prevention-focused DHIs prior to progression to effectiveness or broader implementation trials [48,51].

This feasibility study aligns with the UK MRC framework for the development and evaluation of complex interventions [48], which advocates for early-phase studies to assess feasibility and refine intervention delivery before large-scale implementation or outcome trials. By integrating quantitative and qualitative data, the study will assess not only whether the intervention is used, but also how it is experienced and implemented by both consumers and their clinicians. This mixed methods approach will provide critical insights into the contextual factors that influence intervention success, including barriers and facilitators to engagement and integration in routine care [33,45,46].

The ePREVENT-360 feasibility study has several strengths. It addresses the need to evaluate innovative, person-centered strategies that support proactive health management in general practice [5-7,9-11]. It also adopts an implementation-aware approach that acknowledges the realities of general practice, including time pressures and variable digital readiness [10,12,13]. The design ensures input from consumer and clinician stakeholders across metropolitan, regional, and rural settings, helping to inform future refinement and scaling strategies.

However, the study will have some limitations. As a single-arm feasibility study, it is not designed to assess intervention effectiveness or establish causality. The use of a small, purposively selected sample of 5 clinics, recruited through networks and required to meet minimum staffing criteria, may introduce selection bias, as these sites may not reflect the broader diversity of Australian general practice settings.

Consumer participants will be recruited primarily through convenience sampling and QR-based self-enrollment processes. This approach may preferentially attract participants who are more digitally literate, health-engaged, or socioeconomically advantaged [33,45,46]. It may also result in the underrepresentation of culturally and linguistically diverse communities and people with lower digital literacy. At this stage, THRIVE is available only in English, and developing multilingual intervention materials is neither feasible nor appropriate for this early-phase feasibility study. The purpose of the current trial is to establish core acceptability, usability, and feasibility before investing in broader co-design and multilingual expansion. Finally, while self-reported outcomes may be subject to recall or social desirability bias, these limitations are partially mitigated through the inclusion of objective platform use data.

As a prototype intervention, THRIVE is not yet integrated with general practice EMR systems. The importance of integration and interoperability is well recognized; thus, this limits automation of information transfer. However, it allows for low-disruption implementation during feasibility testing. Planned future development will explore EMR integration, informed by study findings.

Despite these limitations, the ePREVENT-360 will generate critical insights into the feasibility of a novel DHI for chronic condition prevention in general practice. It addresses multiple evidence gaps, including the role of patient activation in digital engagement [21,29] and the practical challenges of embedding DHIs into clinical workflows [37,42,43]. Findings will directly inform intervention refinement and guide the design of a future, powered trial to assess effectiveness and implementation outcomes at scale. This study responds directly to national and global calls for innovative, person-centered prevention strategies and real-time population health indicators and will contribute to the growing field of digital health implementation research [4,5,7,9,36,110].

The study design is informed by the STROBE (Strengthening the Reporting of Observational Studies in Epidemiology) guidelines and the CONSORT (Consolidated Standards of Reporting Trials) 2010 extension to randomized pilot and feasibility trials statement [52,111]. Reporting will adhere to these guidelines, as well as the CRISP (Consensus Reporting Items for Studies in Primary Care) [112]. Results will be disseminated through relevant peer-reviewed journals, conference presentations, and a doctoral thesis.

## Supplementary material

10.2196/83105 Multimedia Appendix 1 ePREVENT-360 study protocol (THRIVE clinical update policy, variables and validation summary, interview guides, and quantitative survey instruments) [66,68-91].

10.2196/83105 Checklist 1 SPIRIT checklist

10.2196/83105 Checklist 2 COREQ checklist.

## Abbreviations

CHAI: Consumer Health Activation Index
CONSORT: Consolidated Standards of Reporting Trials
COREQ: Consolidated Criteria for Reporting Qualitative Research
CRISP: Consensus Reporting Items for Studies in Primary Care
DHI: digital health intervention
EMR: electronic medical record
MRC: Medical Research Council
SAP: statistical analysis plan
SPIRIT: Standard Protocol Items: Recommendations for Interventional Trials
SPSS: Statistical Package for Social Sciences
STROBE: Strengthening the Reporting of Observational Studies in Epidemiology
THRIVE: Tailored Health Risk Insights for Vital Empowerment
UTAUT2: extended unified theory of acceptance and use of technology 2
