# Supplementary material for: Evaluating a Digital Chronic Condition Prevention Intervention (THRIVE) in Australian General Practice: Protocol for a Mixed Methods Feasibility Study (ePREVENT-360)
Source: JMIR Res Protoc. 2026 May 6;15:e83105. doi: 10.2196/83105 (PMC13148338; doi:10.2196/83105)
Supplement: Multimedia Appendix 1 [file resprot-v15-e83105-s001.docx]

ePREVENT-360 protocol : Multimedia Appendix 1

[Supplementary File 1. THRIVE Clinical Content and Algorithmic Update Policy v1.5 2](#_Toc227320793)

[Supplementary File 2. THRIVE variables, validation process and recommendations source summary 4](#_Toc227320805)

[Supplementary File 3. ePREVENT-360 Semistructured Interview Guides 13](#_Toc227320807)

[3.1 ePREVENT-360 consumer qualitative postintervention survey guide 13](#_Toc227320808)

[3.2 ePREVENT-360 clinician qualitative research instruments 14](#_Toc227320810)

[3.2.1 Clinician preintervention semistructured interview guide 14](#_Toc227320811)

[3.2.2 Clinician postintervention semi-structured interview guide 15](#_Toc227320812)

[Supplementary File 4. ePREVENT-360 consumer quantitative research instruments 15](#_Toc227320813)

[4.1 Online quantitative preintervention consumer survey 15](#_Toc227320814)

[4.2. Quantitative online postintervention survey for consumers 19](#_Toc227320815)

[4.3 Quantitative clinician postintervention survey 21](#_Toc227320816)

## Supplementary File 1. THRIVE Clinical Content and Algorithmic Update Policy v1.5

Updated October 25 2025

**Purpose** To ensure that THRIVE’s risk models, preventive recommendations, and educational content remain aligned with the latest Australian preventive health guidelines and evidence, and that updates are made in a timely, transparent and reproducible manner.

## **1. Scope of Updates**

This policy applies to updates to:

- Risk factor inputs
- Weighting logic
- Underlying evidence sources
- Preventive recommendations
- Screening eligibility criteria
- Behavioral-health guidance
- Condition definitions and category thresholds
- Medical-age and vitality-score inputs
- Clinician summary report content (including MBS contextual information)

## **2. Triggers for Updates**

Updates may be triggered by:

### (a) Release of new national guidelines or screening recommendations, including:

- RACGP Red Book
- Cancer Council screening guidelines
- Heart Foundation
- Diabetes Australia
- Kidney Health Australia

### (b) Updates to epidemiological or risk-factor evidence

(e.g., new burden-of-disease estimates, cohort data, risk factor prevalence, new variables such as breast density added following updated recommendations))

### (c) Policy or program changes affecting screening eligibility

(e.g., bowel cancer screening starting age lowering, lung cancer program introduction)

### (d) Internal validation findings

(e.g., need to adjust risk weighting or threshold categories)

### (e) Usability or feasibility study feedback

## **3. Update Frequency**

THRIVE follows a **continuous update cycle** rather than a fixed interval. This means that updates are implemented as soon as practically feasible following guideline changes, rather than waiting 6–12 months or a 10-year guideline cycle. This ensures that consumers and clinicians receive the most current preventive care information.

## **4. Update Process**

Each update follows a structured process:

1. **Evidence Review**
   - Identify guideline or evidence change
   - Assess relevance to conditions included in the THRIVE model
2. **Impact Assessment**
   - Determine which inputs, thresholds or recommendations require modification
   - Evaluate downstream impacts on consumer plans and clinician summaries
3. **Revision of Rulesets / Content**
   - Update logic for risk models, preventive recommendations, or screening criteria
4. **Internal Validation & Quality Chec**
   - Ensure coherent outputs
   - Compare pre-/post-update outputs for consistency
   - Conduct spot-checks against guideline decision tables
5. **Version Control Documentation**
   - Assign release version (e.g., v1.4 → v1.5)
   - Document what changed and why
6. **Deployment**
   - Implement update in production
7. **Communication to Clinicians (as appropriate)**
   - Outline key changes relevant to practice

## **5. Version Control**

Each release includes:

- Version number
- Release date
- Summary of changes
- Evidence/guideline source
- Behavioral logic modified
- Conditions affected

Example of version control entries recently implemented:

- Lung cancer risk: updated to reflect NLSP risk criteria (2024)
- Bowel cancer screening: starting age changed to 45 (2024)
- Breast cancer: incorporation of breast density as a variable (2025)

## 6. Principles Guiding Updates

- **Evidence-aligned**: always reference current, authoritative guidelines
- **Safety-focused**: avoid outdated or incorrect recommendations
- **Rapid translation**: accelerate preventive guideline adoption
- **Transparency**: document rationale and evidence for each change
- **Equity-aware**: update recommendations in line with evolving guidance for priority groups

## Supplementary File 2. THRIVE variables, validation process and recommendations source summary

The table below, summarises the specific risk and protective factors embedded in the THRIVE risk algorithms and how they are operationalized to guide goal setting and care navigation.

|  | Chronic disease lifetime risk | Variables incorporated into lifetime risk algorithm  NB (p) annotates protective factors | Source citations for recommendations provided | Clinical risk calculators and correlation analysis |
| --- | --- | --- | --- | --- |
| 1 | Type 2 diabetes (T2D) and gestational diabetes | **Biometrics:** BMI (relative to ethnicity), waist circumference  **Demographics:** Age, ethnicity (Aboriginal and Torres Strait Islander peoples, Pacific Islander, Middle Eastern, East and South Asian),  **Lifestyle:** Red meat intake, exercise level, smoking status, prolonged sitting at work and sedentary time, sleep time,  **Family history:** T2 diabetes (high- at least 2 first degree on same side or moderate- 1 first degree or 2 second degree)  **Past medical history**: Identified impaired glucose tolerance, polycystic ovarian syndrome, psoriasis, gestational DM or antipsychotic medications.  **Gestational DM specific:** Weight gain in first trimester of pregnancy. | **Current symptoms:** Advised of symptoms of hyperglycemia and advised to have GP review if concerned.  If moderate or high risk, encouraged to access screening in line with 2025 RACGP ‘Red Book’[1] and to address risk factors. | AUSDRISK[2] (5-year risk)  Construct validation process undertaken. The full process is described in a development and validation paper (Authors own, under review))  κ = 0.81, 95% CI 0.75–0.87; ρ=0.87, p<0.001  THRIVE lifetime T2D risk score demonstrated almost perfect ordinal concordance with AUSDRISK-2 demonstrating robust construct validity. |
| 2 | Obstructive sleep apnoea (OSA) | **Biometrics:** BMI (relative to ethnicity) and neck circumference.  **Demographics:** Age and male gender.  **Lifestyle:** Alcohol intake, smoking status  **Family history:** OSA  **Current symptoms:** Falling asleep easily during the day/when driving, snoring, daytime sleepiness and/or witnessed apneas.  **Past medical history**: Hypertension or OSA. | **Current symptoms:** Assessed during assessment.  If high risk encouraged to access a sleep study and address risk factors as per 2025 RACGP ‘Red Book’.[1]  Moderate risk encouraged to address risk factors and to be aware of worsening symptoms. | STOPBANG[3]  Construct validation process undertaken. The full process is described in a development and validation paper (Authors own, under review))  κ= 0.51, 95% CI 0.39-0.64); ρ = 0.67, p<0.001.  A moderate level of ordinal concordance is consistent with expectations as the two instruments measure related but not identical constructs—STOP-BANG identifies current high-risk features whereas THRIVE estimates lifetime risk based on a broader set of variables. |
| 3 | Metabolic Associated Fatty Liver Disease (MAFLD) | No risk score provided. | Recommendations are consistent with the Gastroenterological Society of Australia Recommendations for the assessment of metabolic dysfunction-associated fatty liver disease (MAFLD) in primary care: a consensus statement[4]. Specifically that individuals with BMI≥30 +/- T2D or ≥2 of increased waist circumference relative to ethnicity, systolic BP ≥130 or diastolic BP≥85, or treatment with antihypertensives or TG levels ≥1.7mmol/L or HDL <1.0mmol/L men or <1.3mmol/L women or pre-diabetes are advised to be referred for liver function tests and undertake liver ultrasound. | No widely adopted MAFLD risk prediction tool is currently used in Australian primary care. |
| 4 | Ischaemic heart disease | **Biometrics:** BMI (relative to ethnicity), resting heart rate > 90,  **Demographics:** Age, ethnicity (Aboriginal and Torres Strait Islander peoples, Pacific Islander, Middle Eastern, East and South Asian, African)  **Lifestyle:** Processed meat and sugar intake, fish (p), exercise level, smoking status, prolonged sitting at work, shift work, job strain,  **Family history:** Premature IHD <55y in males, <65y in females  **Current symptoms:** Advised of symptoms of cardiac ischemia and advised to attend for urgent review if experiencing these.  **Past medical history**: Hypertension (mild, mod or severe), diabetes (and recent HbA1C level), obstructive sleep apnoea, CKD, TC/HDL ratio and triglyceride level. | If moderate or high risk encouraged to access screening in line with 2025 RACGP ‘Red Book’[1] and National Heart Foundation of Australia guideline.[5]  Advised of symptoms to be aware of and encouraged to address risk factors. | Aus CV risk calculator 2023[6]  Construct validation process undertaken. The full process is described in a development and validation paper (Authors own, under review))  κ = 0.73, 95% CI 0.65-0.82; ρ = 0.81, p < 0.001  THRIVE lifetime CVD risk demonstrated substantial ordinal concordance with the Australian CVD Risk Calculator. This supports the construct validity of the THRIVE CVD risk model. |
| 5 | Cerebrovascular disease | **Biometrics:** BMI (relative to ethnicity), waist circumference,  **Demographics:** Age, ethnicity (Aboriginal and Torres Strait Islander peoples)  **Lifestyle:** Red meat and alcohol intake, fish (p) and fruit (p) intake, exercise level, smoking status, prolonged work hours, job strain/decision latitude, stress levels and low social support.  **Current symptoms:** Advised of symptoms of TIA and to attend for urgent review if concerned.  **Past medical history**: Atrial fibrillation, hypertension (mild, mod or severe), diabetes (and recent HbA1C level), obstructive sleep apnea, TIA/CVA and valvular heart disease. | If moderate or high risk, encouraged to access screening in line with 2025 RACGP ‘Red Book’[1] and Australian Cardiovascular Disease Risk guideline[5] and to address risk factors. | Aus CV risk calculator 2023[6]  Construct validation process undertaken. The full process is described in a development and validation paper (Authors own, under review))    κ = 0.73, 95% CI 0.65-0.82; ρ = 0.81, p < 0.001  THRIVE lifetime CVD risk demonstrated substantial agreement with the Australian CVD Risk Calculator. This supports the construct validity of the THRIVE CVD risk model. |
| 6 | Hypertension | **Biometrics:** BMI (relative to ethnicity), resting heart rate > 90 and waist circumference.  **Demographics:** Age and ethnicity (Aboriginal and Torres Strait Islander peoples)  **Lifestyle:** Red meat, alcohol and salt intake, exercise level and smoking status, job strain, decision latitude, stress score and perception of job insecurity.  **Past medical history**: Hypertension (mild, mod or severe), diabetes (and recent HbA1C level), obstructive sleep apnoea and CKD. | All individuals encouraged to have blood pressure checked. If moderate or high risk, encouraged to access screening and management in line with 2025 RACGP ‘Red Book,[1] Australian Cardiovascular Disease Risk guideline[5] and to address risk factors. | No widely adopted hypertension risk prediction tool is currently used in Australian primary care. |
| 7 | Chronic kidney disease (CKD) | **Biometrics:** BMI (relative to ethnicity) and resting heart rate > 90  **Demographics:** Age and ethnicity (Aboriginal and Torres Strait Islander peoples)  **Lifestyle:** Alcohol intake, exercise level and smoking status.  **Family history:** CKD  **Past medical history**: Hypertension (mild, mod or severe), diabetes (and recent HbA1C level) and whether aware of previously identified proteinuria, kidney disease, obstructive sleep apnoea and psoriasis. | If moderate or high risk encouraged to access screening in line with 2025 RACGP ‘Red Book’[1], Kidney Health Australia July 2024 CKD guideline[7], Australian Cardiovascular Disease Risk guideline[5] and to address risk factors. | Aus CV risk calculator 2023[6]  Construct validation process undertaken. The full process is described in a development and validation paper (Authors own, under review))  Cohen’s κ= 0.73 (95% CI 0.65-0.82) Spearman’s p 0.81 (p<0.0001)  THRIVE lifetime CVD risk demonstrated substantial agreement with the Australian CVD Risk Calculator. This supports the construct validity of the THRIVE CVD risk model. |
| 8 | Atrial fibrillation (AF) | **Biometrics:** BMI (relative to ethnicity)  **Demographics:** Age and gender (male)  **Lifestyle:** Alcohol intake, exercise level and smoking status.  **Past medical history**: Hypertension (mild, mod or severe), diabetes (and recent HbA1C level), cardiac failure and valvular heart disease. | **Current symptoms:** Advised of symptoms.  If risk factors, high resting heart rate and/or symptoms advised to consult GP (and to use ECG function if they own a smart watch to record ECG if experiencing symptoms). Aligned with Australian guideline recommendations for opportunistic case finding (e.g., pulse check or single-lead ECG in adults ≥65 years).[1] | No widely adopted AF risk prediction tool is currently used in Australian primary care for population-level screening.  The AF risk score[8] was reviewed for construct comparison; however, it is predominantly designed for individuals with established cardiovascular disease and is not routinely applied in general-risk prevention cohorts |
| 9 | Dementia (Alzheimer’s disease and vascular dementias) | **Biometrics:** BMI (relative to ethnicity)  **Demographics:** Age and education level  **Lifestyle:** Vegetable(p) and fish (p)intake, exercise level, smoking status, poor sleep, job strain and low social support.  **Family history:** ≥1 parent diagnosed <80y  **Past medical history**: Hypertension (mild, mod or severe), diabetes (and recent HbA1C level), obstructive sleep apnoea, depression and past history of significant head injury. | If moderate or high risk encouraged to access cardiovascular and diabetes screening in line with 2025 RACGP ‘Red Book’[1] and to address risk factors. | Currently, no dementia risk calculators—including the LIBRA2 index[9]—are recommended within the RACGP Guidelines for Preventive Activities in General Practice.[1] Given the absence of an endorsed screening or risk-stratification tool, the dementia component of our model was informed by modifiable risk factors supported in the prevention literature, with conceptual reference to LIBRA2[9] and ANU-ADRI.[10] Formal comparative validation was not undertaken as these calculators are not broadly used in Australian preventive care. |
| 10 | Mental health:  Depression  Anxiety and  Post traumatic stress disorder | **Biometrics:** BMI  **Lifestyle/work:** Alcohol intake, exercise level, inadequate sleep, job strain, decision latitude, prolonged work hours, shift work, low support at work, prolonged sitting, social isolation, high stress (modified Holmes and Rahe scale), measures of resilience, mindset and purpose.  **Family history:** Depression or anxiety requiring medication or hospitalisation in first degree relative.  **Past medical history:** T2 diabetes  **Past history:** Trauma exposure, family violence. | **Current symptoms:** PTSD symptoms identified within assessment. If symptoms of PTSD, moderate or high risk of depression and/or anxiety are identified individuals are provided link to ‘Are you OK?’ which incorporates a more detailed analysis of symptoms as well as assessment of suicide risk with links to resources and consent for THRIVE clinician to contact them. If there are concerns linkage with professional support is facilitated.  All individuals identified to be at increased risk provided links to relevant online resources including Black Dog Institute, Beyond Blue and Phoenix Australia. | No widely adopted mental health risk prediction tool is currently used in Australian primary care. |
| 11 | Breast cancer | **Biometrics:** BMI(relative to ethnicity) and waist circumference  **Demographics:** Age and ethnicity ( Ashkenazi Jewish and Caucasian)  NB Gender not included to ensure men at high risk are identified.  **Lifestyle/work:** Alcohol and vegetable intake, exercise level, prolonged sitting at work and smoking status  **Family history:** Known BRCA1/BRCA2**,** identified very high, high and moderate risk as per guidelines.  **Past medical history:** Breast cancer, age of menarche, age of menopause, previous pregnancies breastfeeding >5mo and >12mo(p)  **Screening:** Recent mammogram and/or ultrasound (age depends on risk level) and reported high breast density. | Screening recommendations are consistent with Australian guidelines, 2025 RACGP ‘Red Book’ [1] and the national breast cancer screening program.[11]  Individuals are encouraged to address lifestyle risk factors and to understand their risk level to access appropriate screening. | The iPrevent breast cancer risk assessment tool [12] is referenced in the RACGP Preventive Care Guidelines; however, is not recommended for routine use in primary care and requires 30 minutes for completion.[13]  The iPrevent tool, as a validated risk calculator was reviewed for conceptual alignment of risk factors only, but no formal correlation analysis was undertaken. |
| 12 | Bowel cancer | **Biometrics:** BMI(relative to ethnicity)  **Lifestyle/work:** Alcohol, red and processed meat intake, fish intake (p), calcium/dairy (p), fibre (p), exercise level and prolonged sitting at work and smoking status (risk related to pack years including ex-smokers).  **Family history:** Identified very high, high and moderate risk as per guidelines.  **Past medical history:** Bowel cancer, inflammatory bowel disease, polyps  **Screening:** Previous colonoscopy result and routine screening access. | **Current symptoms:** Advised if current symptoms to seek advice from GP.  Screening and risk factor modification recommendations are consistent with Cancer Council 2023 guideline for the prevention, early detection and management of colorectal cancer[14] and the 2025 RACGP ‘Red Book’.[1] | No widely adopted bowel cancer risk prediction tool is currently used in Australian primary care. |
| 13 | Skin cancer (melanoma and non-melanoma skin cancers) | **Biometrics:** Fitzpatrick skin type  **Demographics:** Age  **Lifestyle/work:** Previous solarium exposure, outdoor work with no or minimal protection, propensity to use sun protection.  **Family history:** Identified high risk family history of melanoma.  **Past medical history:** Previous melanoma or NMSC, >100 naevi,  **Screening:** Recent skin check. | **Current symptoms:**  All advised to use sun protection and to be aware of skin changes with link to Cancer Council skin cancer identification resource provided.[15]  Recommendations for melanoma and skin cancer prevention are consistent with national guidelines [1] | The RACGP Preventive Care guidelines [1] recommend melanoma risk assessment based on established risk factors—including age, skin type, personal and family history, sun exposure patterns, and solarium use—rather than through a specific validated risk calculator.  Although melanoma risk prediction tools (e.g., QSkin) have been developed in Australian research contexts, they are not currently integrated into the RACGP Red Book recommendations for routine use in clinical practice.  Accordingly, our model incorporates the guideline-endorsed risk factors and draws on these research tools for conceptual alignment only. |
| 14 | Prostate cancer | **Biometrics:** BMI(relative to ethnicity)  **Demographics:** Age, gender and ethnicity (central and sub-Saharan Africans)  **Lifestyle/work:** Alcohol and red meat intake, exercise level and smoking status.  **Family history:** Identified very high and high risk according to guidelines including identified BRCA1 and BRCA2.  **Past medical history:** Prostate cancer. | **Current symptoms:** All men provided list of symptoms and advised to consult their GP with concerns.  Screening recommendations are consistent with the current Australian guidelines that is, no population screening—except for men at very high risk based on family history of ≥3 close relatives with ≥1 family member <60y—as per 2025 RACGP ‘Red Book’[1].  The RACGP resource for men aged 50-65 years old who were considering prostate cancer screening was previously recommended however is currently being updated as are the national guidelines, following the release of the Prostate Cancer Foundation of Australia draft Clinical Practice Guidelines for the Early Detection of Prostate Cancer in April 2025.[16] The THRIVE recommendations will be revised once the national guidelines and resources are updated. | No widely adopted prostate cancer risk prediction tool is currently used in Australian primary care. |
| 15 | Lung cancer | **Demographics:** Age  **Lifestyle/work:** Smoking status (including current, passive smoker and ex-smoker with pack years calculated), asbestos exposure**,** fruit and vegetable intake and exercise level.  **Family history:** Identified high risk family history. | Screening recommendations are consistent with the Australian Government National Lung Cancer Screening Program guideline July 2025[17] that is, Access to high resolution CT recommended for >30 pack year smokers >50 years of age, with no current symptoms of lung cancer.[17] | No widely adopted lung cancer risk prediction tool is currently used in Australian primary care. |
| 16 | Cervical cancer | No risk score provided but all women asked about screening status and recommended to access screening if appropriate with info on self-collection if preferred. | Recommendations are consistent with the 2025 RACGP ‘Red Book’[1] and Australian national screening program.[11] |  |
| 17 | Pancreatic cancer | No specific risk score provided- determined to be low, moderate or high risk based on:  **Demographics:** Age ≥50  **Biometrics:** High waist circumference/obesity.  **Lifestyle:** High alcohol intake, current smoking.  **Family history:** Identified high risk family history pancreatic cancer and/or BRCA2 trait and/or Ashkenazi Jewish ancestry.  **Personal history:** Diabetes. | The approach to risk identification is consistent with national guidance, which focuses on risk-factor reduction rather than screening or risk-tool–based stratification. The variables included in the Thrive model reflect recognised epidemiological risk factors.  The tool is designed to support lifestyle change and risk awareness rather than estimate absolute cancer risk. Recommendations follow Cancer Australia guidance[18] to focus on primary prevention through modifiable risk reduction as well as prompt review of relevant symptoms or high-risk familial features. | Pancreatic cancer has no established or recommended screening tests, and no validated risk prediction models that are used in routine Australian primary care. |
| 18 | Glaucoma | **Demographics**: Age and ethnicity (African and Asian)  **Family history:** Glaucoma  **Past history:** Myopia  **Screening**: Access to recent tonometry. | Recommended screening is consistent with Optometry Australia guidelines.[19] | No widely adopted glaucoma risk prediction tool is available. |
| 19 | Age-related macular degeneration (ARMD) | **Demographics:** Age >50  **Lifestyle:** Smoking status.  **Family history:** ARMD in a parent or sibling  **Past medical history**: Diabetes (and recent HbA1C result)  **Screening:** Recent eye check in past 2 years if 50-65 years and annual for >65 years. | **Current symptoms:** Advised of symptoms of changes to central vision and need to seek urgent help if this occurs.  Recommended screening is consistent with Optometry Australia guideline.[20]  For people at risk recommend eye checks plus regular Amsler grid self-checks. If high risk and no diagnosis healthy lifestyle recommendations provided. | No widely adopted ARMD risk prediction tool is available. |
| 20 | Osteoporosis | **Biometrics:** BMI (underweight relative to ethnicity)  **Demographics:** Age and gender.  **Lifestyle:** Dairy, high caffeine and alcohol intake, exercise level and smoking status.  **Family history:** Hip fracture <80 years in parent.  **Past medical history**: Previous DXA scan, medications (steroids), premature menopause, low trauma fracture, diabetes (and recent HbA1C result) and chronic kidney disease. | Preventive and screening recommendations are consistent with national osteoporosis guidelines.[21] | For osteoporosis and fracture risk, we reviewed established fracture-risk calculators including FRAX®[22] and the Garvan fracture risk calculator (which underpins the consumer facing ‘Know Your Bones’ tool).[23]  These models estimate 5-10 year fracture probability and are primarily used in older adults to guide treatment decisions rather than primary prevention. The THRIVE score therefore serves a different purpose. There is no widely adopted osteoporosis lifetime risk prediction tool available for comparison. The variables used are conceptually aligned with the FRAX® and the Garvan fracture risk calculators. |
| 21 | Hip and knee osteoarthritis | No risk score provided. Identified as low, moderate or high risk based on:  **Demographics:** BMI (relative to ethnicity) and age  **Lifestyle:** Exercise level  **Family history:** Hip or knee replacement in 1^st^ degree relative ≤65 years | No osteoarthritis risk prediction or screening is recommended in the 2025 RACGP ‘Red Book’.[1] | No osteoarthritis risk prediction tool is widely adopted therefore the OA Risk C calculator[24] was used for conceptual alignment only. |
| 22 | Back and neck pain | **Biometrics:** BMI (relative to ethnicity)  **Lifestyle:** Exercise, prolonged sitting, smoking status,  **Past medical history**: Depression, back pain, job strain, decision latitude, physically demanding role with repetitive tasks, prolonged sitting or standing, high stress levels, perceived low support in workplace. | If moderate or high risk – recommendations provided to improve core stability, maintain regular exercise, safe lifting and to address other risk factors.  There is no back or neck pain risk prediction or screening recommended in the 2025 RACGP ‘Red Book’.[1] | There is no widely adopted chronic back or neck pain risk prediction tool in current use in Australia. |
| 23 | Hepatitis B | No risk score provided.  Recommendation to access screening if risk factors related to country of birth and/or previous IVDU, healthcare worker, close family member or sexual partner diagnosed with hepatitis B or previous prison time. Information on vaccination also provided. | Screening of population groups at high risk is recommended in the 2025 RACGP Red Book and GESA. Australian Consensus Recommendations for the management of Hepatitis B infection.[25] | There is no widely adopted hepatitis B risk prediction calculator in current use in Australia. |

##

##

## REFERENCES

1. Royal Australian College of General Practitioners. Guidelines for Preventive Activities in General Practice. East Melbourne, Victoria: Royal Australian College of General Practitioners,; 2025.

2. Chen L, Magliano DJ, Balkau B, Colagiuri S, Zimmet PZ, Tonkin AM, et al. AUSDRISK: an Australian Type 2 Diabetes Risk Assessment Tool based on demographic, lifestyle and simple anthropometric measures. Med J Aust. 2010;192(4):197-202.

3. Chung F, Abdullah HR, Liao P. STOP-Bang Questionnaire: A Practical Approach to Screen for Obstructive Sleep Apnea. Chest. 2016;149(3):631-8.

4. Gastroenterological Society of Australia (GESA). Recommendations for the assessment of metabolic dysfunction-associated fatty liver disease (MAFLD) in primary care: a consensus statement. Melbourne, Victoria, Australia.; 2024.

5. National Heart Foundation of Australia. Australian Guideline for assessing and managing cardiovascular disease risk. Aus CVD risk calculator and related resources.: National Heart Foundation of Australia,; 2023 [Available from: <https://www.cvdcheck.org.au/overview>.

6. Jennings G, Raffoul N, Nelson M, Australian Guideline For A, Managing Cardiovascular Disease Risk E. Assessing, communicating and managing cardiovascular disease risk: a practical summary of the 2023 guideline. Aust Prescr. 2024;47(2):57-63.

7. Kidney Health Australia. Chronic Kidney Disease (CKD) Management in Primary Care. Melbourne, Australia2024.

8. Brunner KJ, Bunch TJ, Mullin CM, May HT, Bair TL, Elliot DW, et al. Clinical predictors of risk for atrial fibrillation: implications for diagnosis and monitoring. Mayo Clin Proc. 2014;89(11):1498-505.

9. Rosenau C, Kohler S, van Boxtel M, Tange H, Deckers K. Validation of the Updated "LIfestyle for BRAin health" (LIBRA) Index in the English Longitudinal Study of Ageing and Maastricht Aging Study. J Alzheimers Dis. 2024;101(4):1237-48.

10. Hall A, Barbera M, Lehtisalo J, Antikainen R, Huque H, Laatikainen T, et al. The Australian National University Alzheimer's Disease Risk Index (ANU-ADRI) score as a predictor for cognitive decline and potential surrogate outcome in the FINGER lifestyle randomized controlled trial. European journal of neurology. 2024;31(5):e16238.

11. Cancer Council Australia. Cancer Screening Programs: Cancer Council Australia,; NA [Available from: <https://www.cancer.org.au/cancer-information/causes-and-prevention/early-detection-and-screening>.

12. Phillips KA, Liao Y, Milne RL, MacInnis RJ, Collins IM, Buchsbaum R, et al. Accuracy of Risk Estimates from the iPrevent Breast Cancer Risk Assessment and Management Tool. JNCI Cancer Spectr. 2019;3(4):pkz066.

13. Peter MacCallum Cancer Centre. iPREVENT Melbourne, Victoria, Australia.: Peter MacCallum Cancer Centre,; [Available from: <https://www.petermac.org/patients-and-carers/health-services-for-cancer-patients/cancer-prevention/iprevent>.

14. Cancer Council Australia Colorectal Cancer Screening Working Party. Clinical Practice Guidelines for the prevention, early detection and management of colorectal cancer. Sydney, Australia: Cancer Council Australia; 2023.

15. Cancer Council Australia. Check for signs of skin cancer: Get to know your skin, Melbourne, Victoria, Australia: Cancer Council of Australia,; [updated NA. Available from: <https://www.cancer.org.au/cancer-information/causes-and-prevention/sun-safety/check-for-signs-of-skin-cancer>.

16. Prostate Cancer Foundation of Australia 2016 PSA Guideline Review Expert Advisory Panel. DRAFT Guidelines for the Early Detection of Prostate Cancer. Clinical Practice Guidelines for Health Professionals or Public Consultation. . Sydney, Australia.: Prostate Cancer Foundation of Australia,; 2025.

17. Australian Government. National Lung Cancer Screening Program Program Guidelines. Canberra, ACT: Australian Government; 2025 2 August 2025].

18. Australian Government CA. National Pancreatic Cancer Roadmap : Prevention and early detection Canberra: Australian Government,; 2022 [Available from: <https://www.canceraustralia.gov.au/key-initiatives/national-pancreatic-cancer-roadmap/prevention-and-early-detection>.

19. Optometry Australia. Clinical Practice Guide for the Diagnosis and Management of Open Angle Glaucoma. South Melbourne, Victoria: Optometry Australia,; 2020.

20. Optometry Australia. Clinical Practice Guide for the diagnosis, treatment and management of Age-Related Macular Degeneration. South Melbourne, Victoria: Optometry Australia; 2024.

21. The Royal Australian College of General Practitioners and Osteoporosis Australia. Osteoporosis prevention, diagnosis and management in postmenopausal women and men oer 50 years of age. 2nd ed. East Melbourne; 2017.

22. Centre for Metabolic Bone Diseases University of Sheffield. Fracture Risk Assessment Tool Calculation Tool [Available from: <https://www.fraxplus.org/calculation-tool>.

23. Healthy Bones Australia and Garvan Institute of Medical Research. Know your bones: bone health assessment tool NA [Available from: <https://www.knowyourbones.org.au/>.

24. Losina E, Klara K, Michl GL, Collins JE, Katz JN. Development and feasibility of a personalized, interactive risk calculator for knee osteoarthritis. BMC Musculoskelet Disord. 2015;16:312.

25. Hepatitis B Consensus Statement Working Group. Australian consensus recommendations for the management of hepatitis B infection. Melbourne: Gastroenterological Society of Australia; 2022.

##

## Supplementary File 3. ePREVENT-360 Semistructured Interview Guides

## 3.1 ePREVENT-360 consumer qualitative postintervention survey guide

##

| Date of interview: |  |
| --- | --- |
| Start/End time: |  |
| Interviewer: |  |

| Participant’s information |  |
| --- | --- |
| Age : |  |
| Gender : |  |

Thanks for your time today.

Before we start, I’d just like to confirm that you have read, understood and signed the online consent form about this interview? As explained in the study information and consent form, this interview will be recorded. Do you have any questions about that?

I’d like to talk to you about the THRIVE program that you had access to through this study.

1. What initially motivated you to use the THRIVE program?

2. Did you have any concerns about completing a health assessment online?

3. Was there anything you particularly liked or disliked about the THRIVE program?

Prompts: What did you think about the length of the assessment?
 Was anything difficult to understand?

4. Did the THRIVE program influence your health behaviors or decision making at all?

Prompts: How? If yes, have you continued with these changes?

5. Do you have suggestions for improvements to the program?

6. Are you likely to continue to use the THRIVE program?

Probes: Why? Why not?

7. Would you recommend the THRIVE program to family or friends? Why or why not? Prompts: Which groups of individuals are likely to benefit from using the THRIVE program?

Which groups are less likely to benefit?

## 8. Is there anything else you would like to share or discuss?

## 3.2 ePREVENT-360 clinician qualitative research instruments

### 3.2.1 Clinician preintervention semistructured interview guide

| Date of interview: |  |
| --- | --- |
| Start/End time: |  |
| Interviewer: |  |

| **Participant’s information** |  |
| --- | --- |
| Age |  |
| Gender |  |
| Role in clinic |  |
| Years of experience |  |
| Workload (sessions/week and average consultations/day) |  |

Thanks for your time. Before we start, I’d just like to confirm that you have read, understood and signed the online consent form about this interview? As explained in the study information and consent form, this interview will be recorded. Do you have any questions about that?

I'd like to talk to you about primary and preventive care in general practice with you.

1. Are you able to deliver the preventive care you want to, to your patients?
2. What are the main promoters and challenges to delivering the type of care you want in your community?

Prompts: What works well? What doesn’t work so well?

1. How would you like preventive care to be delivered?

Prompts: Are there any additional supports that you need in your clinic?

1. What is needed in the broader health system for quality preventive care?

1. Does digital health have a role in chronic condition prevention?

Prompts: Do you recommend any digital health interventions to your patients?

Do you have concerns about digital health?

1. We are evaluating a digital program that can automate screening for 22 chronic conditions and provide recommendations direct to patients as well as a summary for health providers. What would you need to know before you recommend such a program to your patients?

Prompts: What would make it more or less likely that you would use this intervention?

### 3.2.2 Clinician postintervention semistructured interview guide

| Date of interview: |  |
| --- | --- |
| Start/End time: |  |
| Interviewer: |  |

| **Participant’s information** |  |
| --- | --- |
| Age |  |
| Gender |  |
| Role in clinic |  |
| Years of experience |  |
| Workload (sessions/week and average consultations/day) |  |

Thanks for your time today. Before we start, I’d just like to confirm that you have read, understood and signed the online consent form about this interview? As explained in the study information and

consent form, this interview will be recorded. Do you have any questions abou that?

I’d like to talk to you about your experience of the THRIVE summaries that your patients may have shared with you.

1. Did any of your patients share their THRIVE summaries with you?

Prompts: If so, could you describe your experience of reading through the THRIVE clinical summary? What worked well? What didn’t work so well.

2. Was the preventive clinical decision support information useful?

Prompts: What worked? What didn’t work?

Were the score calculations useful?

3. I'm interested in your thoughts about incorporating digital programs like this routinely into your future workflow. What do you see as the possible impacts?

Prompts: Did patients’ use of the intervention save or add time in consultations?

Could this intervention improve remuneration for general practice clinics?

4. Did you receive any positive or negative feedback from your patients about the intervention?

5. Do you have suggestions for improving the THRIVE program to enhance its effectiveness?

Prompts: To improve remuneration? To improve efficiency of care?

6. Would you like to continue to use the THRIVE program?

Probes: Why? Why not?

## Supplementary File 4. ePREVENT-360 consumer quantitative research instruments

### 4.1 Online quantitative preintervention consumer survey

**BACKGROUND INFORMATION**

Thank you for agreeing to take part in our study. This study aims to explore ways that digital health may be used to help people to prevent chronic conditions and to stay healthy.

Your opinion is important and your responses will be kept confidential and only used for research purposes. We estimate that this survey will take 10-15 minutes to complete.

**To start off we have a few questions about you. None of your personal information will be shared. This information will be used to identify what factors impact whether people choose to use digital health tools or not.**

| Your first name (or preferred name) |  |
| --- | --- |
| Your email address (so that we can send you a link to an online health check and ask for feedback on your experience in a few months’ time) |  |
| The year you were born | Drop down options to select from 1958 to 1993. |
| What is your postcode? | Free text limited to 4 digits |

| Gender at birth | Female | Male | Intersex |
| --- | --- | --- | --- |
| Gender you identify as | Female | Male | Non-binary |

| What is the highest level of education you have completed? | ▢ Started but didn’t finish high school  ▢ High school  ▢ Vocational training  ▢ Tertiary degree  ▢ One or more postgraduate degrees. |
| --- | --- |
| Are you working at the moment? Please select the option that best describes your situation: | ▢ Employed full time  ▢ Employed part time  ▢ Self-employed  ▢ Casual employment  ▢ Unemployed  ▢ Full-time or part-time student |
| Where were you born? Please select the option that best describes your place of birth: | ▢ Australia  ▢ New Zealand  ▢ UK or Ireland  ▢Pacific Islands (PNG, Fiji, Tonga, New Caledonia, Nauru)  ▢ Canada  ▢ United States  ▢ Western or Eastern Europe  ▢ Scandinavia  ▢ South Asia (India, Bhutan, Nepal, Pakistan, Sri Lanka)  ▢ East Asia (China, Japan, Korea, Hong Kong, Macau, Taiwan)  ▢ Southeast Asia (Indonesia, Malaysia, Philippines, Singapore, Thailand, Vietnam, Cambodia, Timor Leste)  ▢ South, East or West Africa (including Somalia, Eritrea, Chad)  ▢ Middle East  ▢ North Africa (Egypt, Morocco, Sudan, Nigeria, Algeria)  ▢ Mediterranean (Greece, Croatia, Italy, Spain)  ▢ South or Central America |
| What is your ethnicity?  This may not be an easy question for some families. If your parents or grandparents come from different countries please choose the first option that best represents your family’s background: | ▢ First Australian, Aboriginal or Torres Strait Islander  ▢ Pacific Islands (PNG, Fiji, Tonga, New Caledonia, Nauru)  ▢ South Asia (India, Bhutan, Nepal, Pakistan, Sri Lanka)  ▢ East Asia (China, Japan, Korea, Hong Kong, Macau, Taiwan)  ▢ Southeast Asia (Indonesia, Malaysia, Philippines, Singapore, Thailand, Vietnam, Cambodia, Timor Leste)  ▢ South, East or West Africa (including Somalia, Eritrea, Chad)  ▢ Middle East, North Africa (Egypt, Morocco, Sudan, Nigeria, Algeria)  ▢ Mediterranean (Greece, Croatia, Italy, Spain)  ▢ South or Central America  ▢ Native American or Canadian First Nations  ▢ Caucasian  ▢ Other ______________________________ |

| Next, we have a few questions about your health and whether you’ve had a check-up recently. | Excellent | Very good | OK | Poor | Very poor |
| --- | --- | --- | --- | --- | --- |
| In general, how healthy do you feel? |  |  |  |  |  |

|  | Less than once a year | 1-3 visits a year | 4-5 visits a year | 6-11 visits a year | 12-19 visits a year | 20 or more visits per year |
| --- | --- | --- | --- | --- | --- | --- |
| On average, how often do you visit your GP or nurse? |  |  |  |  |  |  |
|  | Never had a check up | More than 5 years ago | Between 2 and 5 years ago | Between 1 and 2 years ago | Within the past year |  |
| How long has it been since you last visited a doctor for a routine check-up? |  |  |  |  |  |  |

| **We’d like to ask you about your experience of chronic conditions. These include high blood pressure, high cholesterol, diabetes, heart and kidney disease, any type of cancer, osteoporosis and sleep apnoea.** | Yes | No | I’m not sure |
| --- | --- | --- | --- |
| Have you ever spoken to your GP or nurse about your risk of chronic conditions? |  |  |  |
| Have you been diagnosed with a chronic condition? |  |  |  |
| Do you have any close friends or family who have been affected by a chronic condition? |  |  |  |

| **Can you rate how strongly you agree or disagree with each of the following:** | Strongly agree | Agree | Somewhat agree | Neither agree or disagree | Somewhat disagree | Disagree | Strongly disagree |
| --- | --- | --- | --- | --- | --- | --- | --- |
| I am aware that my health could be improved. |  |  |  |  |  |  |  |
| I am concerned about my risk of a chronic condition. |  |  |  |  |  |  |  |
| I am concerned about my life being cut short by a chronic condition. |  |  |  |  |  |  |  |

| **We’re interested in how you think about your health and how much control you feel that you have over it at the moment.**    Can you rate how strongly you agree or disagree with each of the following: | Strongly agree | Agree | Slightly agree | Slightly disagree | Disagree | Strongly disagree |
| --- | --- | --- | --- | --- | --- | --- |
| It is very important that I treat my health as a top priority. |  |  |  |  |  |  |
| I always know what steps to take when I have a health problem. |  |  |  |  |  |  |
| I always know how to make myself feel better. |  |  |  |  |  |  |
| I always know where to look for information before making decisions about my health. |  |  |  |  |  |  |
| I can always take care of myself. |  |  |  |  |  |  |
| It is very easy for me to understand my doctor’s or nurse’s instructions. |  |  |  |  |  |  |
| It is very easy for me to make changes to my daily life to improve my health. |  |  |  |  |  |  |
| It is very easy for me to follow my doctor’s or nurse’s instructions. |  |  |  |  |  |  |
| I always attend all of my health appointments. |  |  |  |  |  |  |
| I always make the health changes I should even if I don’t feel well. |  |  |  |  |  |  |

| **Can you tell us a little bit about your relationship with your GP?**  Just a reminder that no information that can identify you will be shared.  Can you rate how strongly you agree or disagree with each of the following: | Strongly agree | Agree | Neither agree or disagree | Disagree | Strongly disagree |
| --- | --- | --- | --- | --- | --- |
| My GP is extremely thorough and careful |  |  |  |  |  |
| My GP cares more about what is convenient for them that my medical needs.* |  |  |  |  |  |
| I completely trust my GPs decisions about which medical treatments are best for me. |  |  |  |  |  |
| My GP is totally honest in telling me about the different treatment options available for my condition. |  |  |  |  |  |
| I have complete trust in my GP. |  |  |  |  |  |

Many thanks for taking part. You will be sent a link to the THRIVE online ‘Health Check’ soon.

*NB These questions are reverse scored

### 4.2. Quantitative online postintervention survey for consumers

**BACKGROUND INFORMATION**

Thank you for being a part of the ePREVENT-360 research project.

Your feedback is very important in shaping future ways to keep Australians healthy. We’re interested in your thoughts whether you chose to use the THRIVE program or not.

Any information you provide will be confidential. We estimate that this survey will take between 1 and 10 minutes.

| **We have a few questions about your health.** | Excellent | Very good | OK | Poor | Very poor |
| --- | --- | --- | --- | --- | --- |
| In general, how healthy do you feel? |  |  |  |  |  |

| **We’re interested in how you think about your health and how much control you feel that you have over it at the moment.**    Can you rate how strongly you agree or disagree with each of the following: | Strongly agree | Agree | Slightly agree | Slightly disagree | Disagree | Strongly disagree |
| --- | --- | --- | --- | --- | --- | --- |
| It is very important that I treat my health as a top priority. |  |  |  |  |  |  |
| I always know what steps to take when I have a health problem. |  |  |  |  |  |  |
| I always know how to make myself feel better. |  |  |  |  |  |  |
| I always know where to look for information before making decisions about my health. |  |  |  |  |  |  |
| I can always take care of myself. |  |  |  |  |  |  |
| It is very easy for me to understand my doctor’s or nurse’s instructions. |  |  |  |  |  |  |
| It is very easy for me to make changes to my daily life to improve my health. |  |  |  |  |  |  |
| It is very easy for me to follow my doctor’s or nurse’s instructions. |  |  |  |  |  |  |
| I always attend all of my health appointments. |  |  |  |  |  |  |
| I always make the health changes I should even if I don’t feel well. |  |  |  |  |  |  |

| **Can you share your thoughts on the Thrive program?** | Yes | No | I’m not sure |
| --- | --- | --- | --- |
| Did you complete the Thrive online Health Check? (if no logic jump to next section) |  |  |  |
| Did you receive an Action Plan? |  |  |  |
| Did you access your Thrive dashboard? |  |  |  |

| **Can you tell us how strongly you agree or disagree with the following statements:** | Strongly agree | Agree | Somewhat agree | Neither agree or disagree | Somewhat disagree | Disagree | Strongly disagree |
| --- | --- | --- | --- | --- | --- | --- | --- |
| I found the Thrive program to be useful |  |  |  |  |  |  |  |
| Using the Thrive program increased my chances of staying healthy. |  |  |  |  |  |  |  |
| Using the Thrive program helped me to accomplish my health goals more quickly. |  |  |  |  |  |  |  |
| Learning how to use the Thrive program was easy for me. |  |  |  |  |  |  |  |
| People who are important to me thought that I should use the Thrive program. |  |  |  |  |  |  |  |
| People whose opinions I value thought I should use the Thrive program. |  |  |  |  |  |  |  |
| I have the resources I needed to use the Thrive program. |  |  |  |  |  |  |  |
| I have the knowledge needed to use the Thrive program. |  |  |  |  |  |  |  |
| I know how to access technical support to use the Thrive program. |  |  |  |  |  |  |  |
| Using the Thrive program was interesting. |  |  |  |  |  |  |  |
| Using the Thrive program was enjoyable. |  |  |  |  |  |  |  |
| The use of the Thrive program has become a habit for me. |  |  |  |  |  |  |  |
| I intend to continue using the Thrive program in the future. |  |  |  |  |  |  |  |
| I trust the Thrive program. |  |  |  |  |  |  |  |
| I found the Thrive program provided reliable information. |  |  |  |  |  |  |  |
| I believe the Thrive program is a safe way to improve my health. |  |  |  |  |  |  |  |
| I trust that the Thrive program will help me to stay healthy. |  |  |  |  |  |  |  |
| The time taken to use the Thrive program was reasonable. |  |  |  |  |  |  |  |

| **What motivated you to start the Health Check? Choose as many as you like:** | ▢ I wanted to improve my health.  ▢ My doctor recommended it.  ▢ I was interested to see my health scores.  ▢ It was recommended by someone I know.  ▢ I wanted to see my personal Action Plan.  ▢ I wanted a gift card.  ▢ Other _____________________________________ |
| --- | --- |

| **Can you share the reasons why you decided not to use the Thrive program?**  Please rate how much you agree or disagree with the following statements: | Strongly agree | Agree | Somewhat agree | Neither agree or disagree | Somewhat disagree | Disagree | Strongly disagree |
| --- | --- | --- | --- | --- | --- | --- | --- |
| I know what I need to do to stay healthy so I didn’t need this. |  |  |  |  |  |  |  |
| There was too much information and I found it overwhelming. |  |  |  |  |  |  |  |
| The technology was difficult to work out. |  |  |  |  |  |  |  |
| The time required to use the Thrive program was reasonable.* |  |  |  |  |  |  |  |
| I don’t like using online health tools. |  |  |  |  |  |  |  |
| The information was difficult to understand. |  |  |  |  |  |  |  |
| I trust the Thrive program.* |  |  |  |  |  |  |  |
| I don’t feel safe putting my personal information online. |  |  |  |  |  |  |  |
| People who are important to me said I should use the Thrive program. |  |  |  |  |  |  |  |
| People whose opinions I value thought I should use the Thrive program. |  |  |  |  |  |  |  |
| I know how to access technical support to use the Thrive program. |  |  |  |  |  |  |  |
| I intend to use the Thrive program in the future.* |  |  |  |  |  |  |  |

| **We’d love your ideas on what could make the Thrive program better. Below is a list of things that could be included in the Thrive program in future.**  Can you rate how useful each of these things would be to you? | Highly useful | Very useful | Moderately useful | Slightly useful | Not at all useful |
| --- | --- | --- | --- | --- | --- |
| Connecting with data from wearables (e.g. smartwatch steps and sleep patterns). |  |  |  |  |  |
| A phone app where I could update my information, recalculate my scores and see my outstanding actions. |  |  |  |  |  |
| Access to online referrals that I need. |  |  |  |  |  |
| A virtual health coach to help me achieve my goals. |  |  |  |  |  |
| Regular updates on health topics that I’m interested in. |  |  |  |  |  |
| Online health and wellness support communities. |  |  |  |  |  |

| Is there anything else you’d like to tell us about your experience or do you have any other ideas about how Thrive could be improved? (short answer question) |  |
| --- | --- |

We’re very grateful that you have taken the time to complete this survey. If you have any further comments or questions, please contact Dr Gillian Singleton at XXXX

*NB These questions are reverse scored.

### 4.3 Quantitative clinician postintervention survey

Thank you for being a part of the ePREVENT-360 research project.

THRIVE is designed to improve healthy life expectancy for patients, reduce workload and improve remuneration for general practice clinics. We’d value your feedback on your experience with the THRIVE program, specifically the clinical summary which included chronic disease prevention recommendations that your patients may have shared with you.

We’re interested in both what worked well and what could be improved.

We estimate that this will take 5 minutes to complete.

|  | <5 | 5-10 | 11-20 | 21-50 | 51-100 | >100 |
| --- | --- | --- | --- | --- | --- | --- |
| **How many of your patients would you estimate, shared their THRIVE prevention summary with you?**: |  |  |  |  |  |  |

| **Can you tell us how strongly you agree or disagree with the following statements about the THRIVE chronic disease risk summary:** | Strongly agree | Agree | Somewhat agree | Neither agree or disagree | Somewhat disagree | Disagree | Strongly disagree |
| --- | --- | --- | --- | --- | --- | --- | --- |
| The THRIVE clinical summary and recommendations were useful. |  |  |  |  |  |  |  |
| It saved me time in consultations. |  |  |  |  |  |  |  |
| It helped me to motivate my patients to make healthier choices. |  |  |  |  |  |  |  |
| There was no new information presented as I already knew my patient’s risk of chronic conditions. * |  |  |  |  |  |  |  |
| It helped me to motivate my patients to access recommended screening. |  |  |  |  |  |  |  |
| It helped me to bill additional MBS item numbers. |  |  |  |  |  |  |  |
| I found the summary was too long*. |  |  |  |  |  |  |  |
| It helped me to work more efficiently with the clinic GPs or nurses. |  |  |  |  |  |  |  |
| I didn’t have time to read the summary properly*. |  |  |  |  |  |  |  |
| Doctors or nurses I work with recommended that I use it. |  |  |  |  |  |  |  |
| I trusted the risk information and recommendations that were provided. |  |  |  |  |  |  |  |
| It wasn’t useful as I had already calculated my patients’ chronic disease risk scores.* |  |  |  |  |  |  |  |
| It provided me with useful insights and information to deliver more effective preventive care. |  |  |  |  |  |  |  |
| Support was available if I had questions about the summary. |  |  |  |  |  |  |  |
| I would recommend the THRIVE program to my patients in future. |  |  |  |  |  |  |  |

| Do you have any other feedback on the THRIVE chronic condition risk summary? |  |
| --- | --- |
| Is there anything else you’d like to tell us about your experience or do you have any other ideas about how THRIVE could be improved for clinicians? |  |

We’re very grateful that you have taken the time to complete this survey. If you have any further comments or questions, please contact XXXXX at XXXXX

*NB These questions are reverse scored.
