# Supplementary figures and images for: Evaluating a Digital Chronic Condition Prevention Intervention (THRIVE) in Australian General Practice: Protocol for a Mixed Methods Feasibility Study (ePREVENT-360)
Source: JMIR Res Protoc. 2026 May 6;15:e83105. doi: 10.2196/83105 (PMC13148338; doi:10.2196/83105)

## Consolidated Criteria for Reporting Qualitative Research (COREQ) checklist


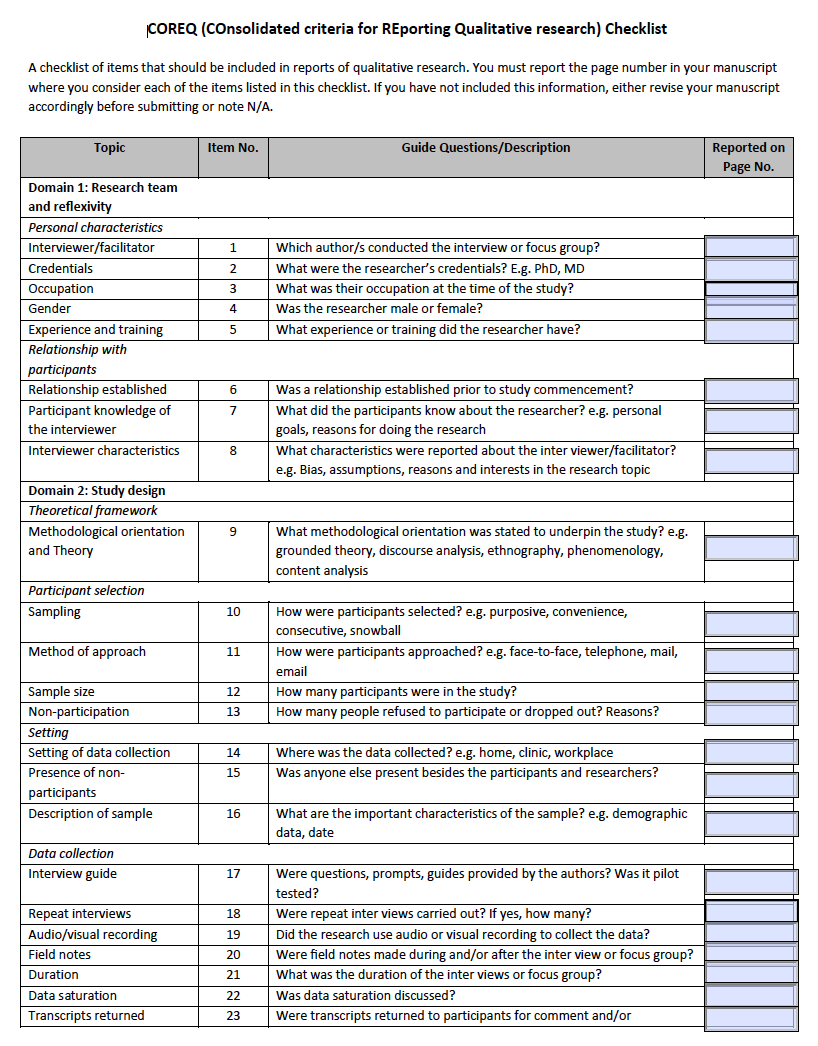


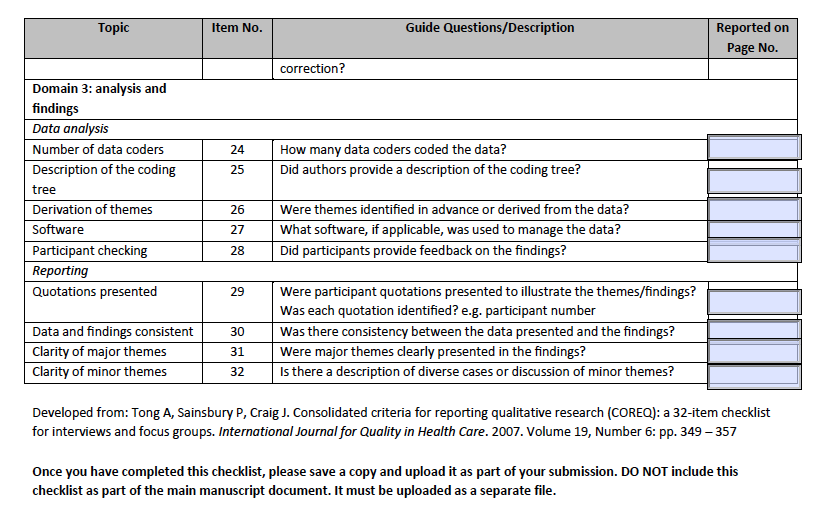

Supplement: Checklist 2 [file resprot-v15-e83105-s003.docx]
